# Comparison of different semi-automated cfDNA extraction methods in combination with UMI-based targeted sequencing

**DOI:** 10.18632/oncotarget.27183

**Published:** 2019-10-01

**Authors:** Anna Streubel, Albrecht Stenzinger, Susann Stephan-Falkenau, Jens Kollmeier, Daniel Misch, Torsten Gerriet Blum, Torsten Bauer, Olfert Landt, Alexander Am Ende, Peter Schirmacher, Thomas Mairinger, Volker Endris

**Affiliations:** ^1^ Department of Pathology, Helios Klinikum Emil von Behring, Berlin, Germany; ^2^ Institute of Pathology, University Hospital Heidelberg, Heidelberg, Germany; ^3^ German Cancer Consortium (DKTK), Heidelberg, Germany; ^4^ Department of Pneumology, Helios Klinikum Emil von Behring, Berlin, Germany; ^5^ TIB Molbiol Syntheselabor GmbH, Berlin, Germany

**Keywords:** lung cancer, liquid biopsy, cfDNA, molecular diagnostics, T790M

## Abstract

The analysis of circulating cell-free DNA (cfDNA) extracted from peripheral blood can serve as a minimally invasive alternative to tumor tissue biopsies in cases with impaired access to tissue. Its clinical utility has been well demonstrated for EGFR T790M testing in lung cancer patients suffering progress after tyrosine kinase inhibitor treatment. At present, highly sensitive unique molecular identifiers (UMI)-based NGS for liquid biopsy testing is less established compared to single gene assays. However, the critical bottleneck are sufficient cfDNA yields, which are essentially required to obtain meaningful test results.

We compared four different cfDNA extraction methods (Qiagen, Promega, Thermo and Stratec) using the same plasma samples in order to evaluate their suitability for further NGS analysis. We managed to draw 60 ml blood from 12 patients each and equally collected 30ml in PAXgene and EDTA tubes at the same time point, sufficient for total of 96 cfDNA extractions. CfDNA concentrations and total amounts were highest for Qiagen and Promega protocols, showing the best read length profiles after sequencing.

Known oncogenic driver mutations were identified in 9 out of 12 patients with at least one of the cfDNA extraction methods, again favoring the extraction protocols from Qiagen and Promega. We also uncovered putative sequencing artefacts including known driver genes pointing to a careful consideration for the limit of detection of this methodology. Our study shows that pre-analytical optimization is necessary to achieve the maximum sensitivity of UMI-based sequencing but also highlights the low abundance of tumor-derived cfDNA in lung cancer samples.

## Introduction

In oncology, the continuously evolving concept of precision medicine is mainly based on the development of drugs that are specifically tailored to address the molecular genetic driver mutation responsible for oncogenic progress [1, 2]. To detect and monitor these genetic changes, various molecular methods have been implemented in routine diagnostics, most of them working with PCR-based techniques.

Up to the very recent present, nucleotides were extracted from fresh or fixed tumor tissue samples. In most cases formalin-fixed and paraffin embedded (FFPE) tumor tissue is available for mutation analysis. Many studies have been carried out to optimize pre-analytical steps and furthermore broaden the spectrum of molecular analyses that can be utilized in this type of material. The main obstacles with FFPE-derived nucleotide acids are their relatively low quality due to fixation-induced degradation and deamination processes that can lead to false-positive sequencing results. While careful considerations of pre-analytical steps minimize these problems, the availability of tumor tissue is restricted to clinical cases where invasive tumor sampling is not limited by the physical fitness of the patient and/or the tumor location.

The concept of DNA analysis without the need of invasive surgical procedures has gained attention and holds reasonable advantages, especially in tumor patients where tumor tissue is not easily available. This is particularly true for monitoring of treatment response in malignant tumors, where repeated gaining of tumor tissue is seriously limited.

A pioneer in routine use of mutation analysis out of plasma has been the detection of the T790M resistance mutation in *EGFR*-mutated NSCLC samples. On the background of increasingly sensitive and specific DNA detection methods, cell free DNA (cfDNA) gained attention and the fascinating concept of detection of genetic alterations by analyzing this source of DNA from easily accessible blood plasma came into focus [3–8].

Although this approach is highly elegant and the mutation looked for is exactly defined, surprisingly the method has not revolutionized the diagnostic procedure. This may be due to the fact that results obtained in many laboratories are disappointing, as especially in NSCLC patients cell-free tumor DNA concentrations are critically low [5] making successful analysis very challenging even when highly sensitive detection methods are applied [9]. Variant allele frequencies (VAF) of mutations often range below 1%, which is below the detection threshold of currently used technologies for mutation analysis using FFPE material such as Sanger sequencing, most real-time PCR or even NGS. To analyse cfDNA several approaches have been developed ranging from single PCR assays to whole genome sequencing [10–12]. Some of these methods reach a theoretical limit of detection (LOD) down to 0.00025-0.01% VAF [10, 13]. For NGS-based approaches this was achieved by introduction of unique molecular identifiers (UMI) to each single DNA molecule allowing deduplication with the downside for the need of ultra-deep sequencing [14, 15]. UMI features some decent advantages compared to standard NGS protocols and compared, to single gene assays UMI-based NGS enables multigene analysis for the detection of resistance mechanisms other than the classical T790M mutation.

Nevertheless the findings of T790M and its primary mutation in cfDNA gained from plasma remain disappointingly low compared with the tissue bound analysis. One of the causes may be found in the pre-analytical procedures used.

To achieve sufficient cfDNA yields, a careful selection of pre-analytical methods is mandatory [6, 7, 8, 16]. This includes not only the choice of blood containers (stabilizing tubes vs. standard EDTA [17]) and transport conditions, but especially also the choice of cfDNA extraction methods.

Hence, extraction methods that yield sufficient amounts of cfDNA molecules matching sequencing coverage are paramount to reach theoretical sensitivity levels. However, data investigating the performance and impact of different extraction methods are very limited. In this context one of the very challenging issues is the availability of sufficient sample volume /plasma with NSCLC patients especially [6, 7]. In this study our aim was to i) evaluate and standardize pre-analytical procedures with the ambition to ameliorate the detection rates of relevant genomic aberrations from circulating cfDNA gained from 4ml plasma input per extraction and ii) evaluate a highly-sensitive NGS-based approach that might overcome some of the restrictions of currently used cfDNA analysis strategies.

## Results

### Study concept

In our cohort of 12 patients, six patients had initial diagnosis of NSCLC at different stages (stage IIA-IV) without any prior cancer therapy (samples P1-P6, Table 1) and blood was taken before therapy, in case of P1, the only one with a stage II situation, blood was collected after surgery. Patients were selected for this study when molecular testing of corresponding FFPE tissue revealed an activating *EGFR* (five patients) or *KRAS* (one patient) mutation (Table 1 and Figure 4). The remaining six patients in this study progressed after first-line tyrosine kinase inhibitor (TKI) therapy targeting an initially identified activating *EGFR* mutation (samples S1-S6, Table 1).

**Table 1:** Patient details and therapy regiment

**ID**	age	gd	st	**FD**	FFPE collection	Mutation (FFPE)	blood coll.	time from FD (d)	blood coll.	first-line/second-line
**P1**	81	f	IIA	02.05.2017	02.05.2017	EGFR:p.Leu858Arg	10.05.2017	8	-	Surgery, no adjuvant chemotherapy
**P2**	74	m	IV	08.05.2017	08.05.2017	EGFR:p.Glu746_Ala750del TP53:p.Leu257Arg PTEN:p.Ser170fs*13	12.05.2017	4	before therapy	palliative brain-RTX + Afatinib until 10/2017
**P3**	74	m	IIIB	28.04.2017	28.04.2017	EGFR:p.Glu746_Ala750del TP53:p.Tyr220Cys	19.05.2017	21	after therapy	Simultanous radiotherapy/chemotherapy (Platin/VRB)
**P4**	69	m	IV	26.05.2017	26.05.2017	EGFR:p.Glu746_Ala750del	30.05.2017	4	before therapy	Erlotinib ± Ramucirumab in study, since 05/2017 Erlotinib monotherapy
**P5**	78	m	IV	31.05.2017	31.05.2017	EGFR:p.Leu747_T751del	01.06.2017	1	before therapy	since 06/2017 Afatinib
**P6**	66	m	IV	04.05.2017	04.05.2017	KRAS:p.Gly13Cys STK11:p.Val197fs*69	10.05.2017	6	before therapy	palliative chemotherapy cisplatin/VRB->Atezolizumab->Pembrolizumab->Tarceva
**S1**	74	m	IV	07.11.2016	07.11.2016	EGFR:p.Leu858Arg	16.05.2017	190	after therapy	TKI (Erlotinib) until 06/2017, restaging SCLC
**S2**	86	f	IV	01.04.2016	01.04.2016	EGFR:p.E746_A750delinsQP	19.05.2017	413	after therapy	Gefitinib until 04/2017, 06-10/2017 Osimertinib
**S3**	81	f	IV	10.07.2013	10.07.2013	EGFR:p.Leu858Arg EGFRR:p.T790M	19.05.2017	1409	after therapy	07/2013-05/2017 Erlotinib, 06/2017 Osimertinib
**S4**	68	f	IV	04.09.2015	04.09.2015	EGFR:p.Glu746_Ala750del	20.07.2016	320	after therapy	09/2015-07/2016 Erlotinib, 07/2016-06/2017 Osimertinib, Chemotherapy
**S5**	84	m	IIIA	01.11.2010	01.11.2010	EGFR:p.Glu746_Ala750del	31.07.2017	2464	after therapy	neoadjuvant Gefitinib, then radio-/chemotherapy + surgery + adjuvant chemotherpy
**S6**	83	f	IV	23.03.2016	23.03.2016	EGFR:p.L861Q PIK3CA:p.Glu545Lys TP53:p.Ser166*	30.05.2017	433	after therapy	05/16-06/17 Erlotinib, then no further therapy

Abbreviations:

ID: Sample Identification; m: male; f: female; FD: first diagnosis; RTx: radiotherapy; SCLC: squamous cell carcinoma; VRB: Vinorelbin; TKI: Tyrosine Kinase Inhibitor; blood coll.: blood collection; gd: gender; st: stage.

**Figure 1:**
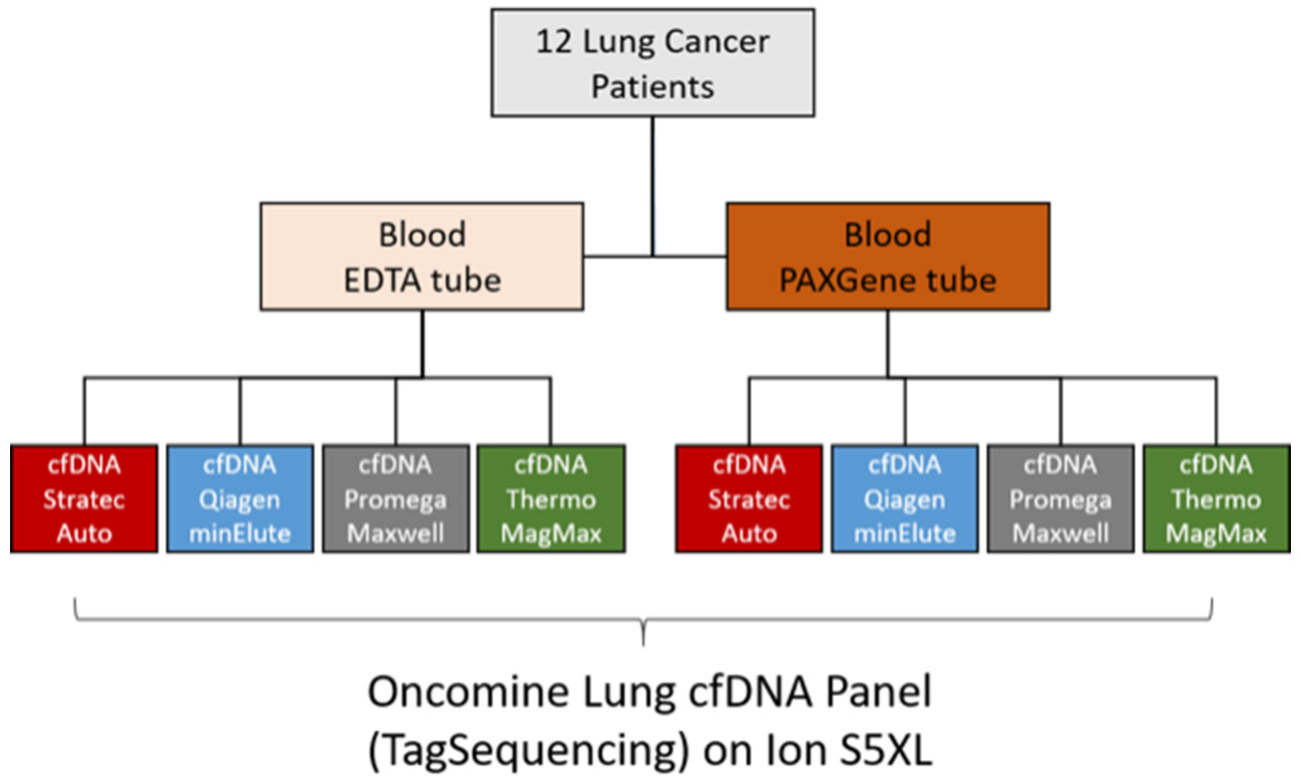
Workflow overview. From all lung cancer patients, blood was collected on the same day to ensure comparability of the results. Independent of the storage vessel (EDTA or PAX), plasma was separated within one hour after blood collection to ensure optimal results. After cfDNA extraction, NGS libraries were prepared using TagSequencing technology and sequenced on an IonTorrent S5 sequencer.

Blood was equally harvested in standard EDTA tubes and PAXgene tubes to determine any different influences of the two blood stabilizing containers on cfDNA extraction and high-sensitive mutation detection. A summary of the study concept is depicted in Figure 1.

### Comparison of cfDNA extraction methods

For our comparison study, we selected four commercially available (semi-)automated cfDNA extraction protocols. All four tested methods allow an input of up to 4 ml of plasma, in 80 of 96 extractions 4ml plasma was available (see Supplementary Table 1). Table 2 summarizes the four protocols: The Stratec protocol provided the highest level of automation operating fully automated with hands-on time of 5 minutes but has a long run time of 160 min, while all other protocols required manual pre-processing of plasma samples prior to samples processing on the robotic machines (Table 2). In case of low sample throughput yet with a need for rapid reporting at the same time one clear technical disadvantage observed with the extraction kits used from Stratec is shown in suboptimal efficiency of reagents coverage if not 12 samples are run simultaneously. A technical disadvantage of 60 min hands-on time show the Thermo and Maxwell protocols compared to Stratec and Qiagen with 5/30 min, respectively. Considering these technical issues, the Qiagen protocol shows a summary of advantages in the least hands-on time combined with a maximum reagent coverage and highest elution volume and number of sample processing flexibility. For this study, elution volumes varied between 36 µl (Qiagen) and 70 µl (Stratec), even though the elution volume can be lowered down to 15 µl using the QiaCube instrument from Qiagen.

**Table 2:** Overview of extraction methods

manufacturer	Promega	Thermo Scientific	Qiagen	Stratec Molecular
**robot**	Maxwell® RSC	KingFisher™ Flex	QIAcube System	InviGenius plus
**product**	RSC LV ccfDNA Kit,	MagMAX™ Cell-Free DNA Isolation Kit	QIAamp® MinElute® ccfDNA	InviMag® Free Circulating DNA Kit/ IG
**type**	bead based	bead based	filter based	bead based
**sample volume (mL)**	1-4; in this study 4	1-5; in this study 4	1-4; in this study 4	4; in this study 4
**elution volume (µl)**	60	50-100; in this study 50	15-60; in this study: 40	125
**recoverable elution volume (µl)**	~ 45, in this study 45	~ 35-80, in this study 45	10-55, in this study: 36	70-80; in this study: 70
**level of automation**	semi	semi	semi	full
**manual pre processing**	external proteinase K step, concentration of DNA from the plasma with magnetic particles in 15mL tubes	external proteinase K step; manual pipetting of the deep well plates	external proteinase K step; concentration of DNA from plasma with magnetic particles in 50 mL tubes	none
**hands-on time (minutes)**	~60	~60	~30	~5
**automated runtime (minutes)**	~35	~35	~25	~160
**total runtime (minutes)**	~95	~95	~55	~165

### cfDNA quantity

**Figure 2:**
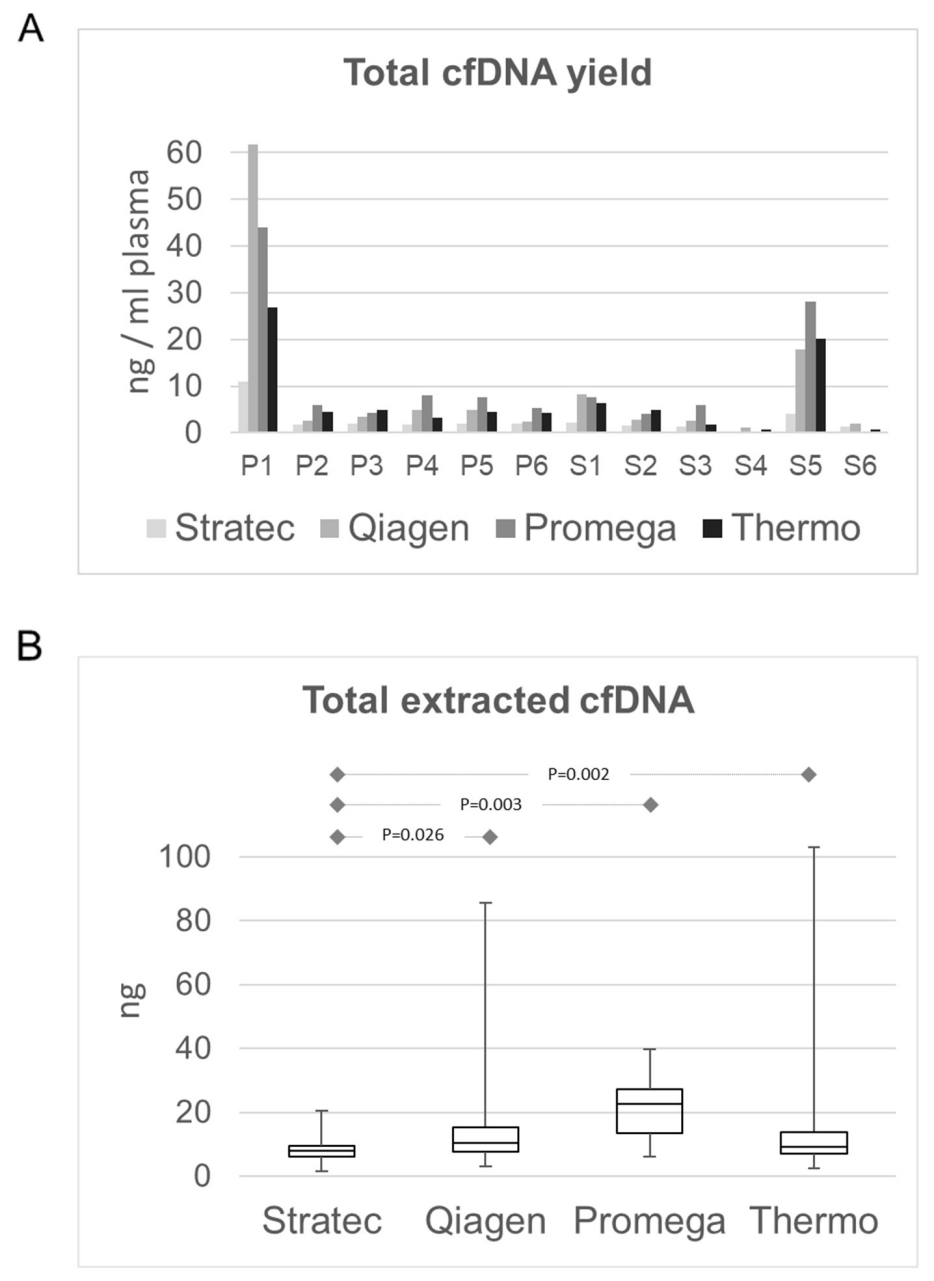
Comparison of total extracted cfDNA amounts. **(A)** cfDNA amount in ng/ml plasma per sample and extraction method. With the exception of sample P1 and sample S5, the available cfDNA concentrations for further library preparations were below 0.5 ng/µl. **(B)** cfDNA yields as box-and-whisker plots from the different extraction protocols. The highest yields were obtained from the Promega MaxWell protocol. While the cfDNA amounts from the Qiagen and Thermo protocols were comparable, the Stratec instrumentation yielded the lowest cfDNA amounts.

In a first step, we compared the total cfDNA concentrations that were obtained by the four protocols. Most samples had cfDNA concentrations independent of the different extraction protocols below 0.5 ng/µl and a mean total amount of extracted cfDNA of 11.5 ng. (Figure 2A+B). Only two samples (P1 and S5) had higher amounts, ranging up to 282 ng in sample P1. The latter sample P1 derived from a patient whose blood was drawn shortly after surgical tumor resection. The highest DNA quantities were obtained using the Promega Maxwell protocol with a mean cfDNA amount of 24.7 ng using 4 ml of plasma (range 12.6 - 188.4 ng), corresponding to a mean of 6.3 ng/ml plasma (Figure 2B). Thermo and Qiagen yielded similar values, with a mean of 9.4 ng (Thermo; range 2.5-132.5ng, mean 4.0 ng/ml plasma) and 10.8 ng (Qiagen; range 3.1 – 282.8 ng, mean 3.4 ng/ml plasma), respectively. The lowest cfDNA amounts were obtained using the Stratec protocol (mean 8.0 ng, range 1.4 – 53.4 ng, mean 1.7 ng/ml plasma). While difference in cfDNA amounts were not statistically significant between the protocols of Qiagen, Promega and Thermo, the difference of each of these methods compared to the Stratec extraction was (paired t-test: Stratec/Qiagen: p=0.0263; Stratec/Promega: p=0.0031, Stratec/Thermo: p=0.0023). Comparing the total extracted cfDNA yields, there was a significant higher cfDNA fraction from plasma stored in EDTA versus PAXgene tubes (paired test: p=0.000097).

Due to the very low cfDNA concentrations, length profile assessment by using fragment analysis on a TapeStation was inconclusive. Supplementary Figure 1 illustrates read profiles for sample S1, showing similar patterns of cfDNA from all methods, with typical peaks at approximately 175bp and 353bp. As exemplified with sample P5, the read profiles for the different other cfDNA extractions were too low to draw any conclusions.

### NGS library preparation

In total, 96 NGS libraries were prepared (eight extractions per sample from 12 samples all together). Using the most possible input of 13 µl, the DNA input for all but patient P1 and S5 was below 20 ng. Library concentrations ranged from 22 to 323 pM (Supplementary Figure 1). Six out of 96 libraries failed QC criteria; e.g. the library concentrations were below the optimal concentration of 50pM (P3 Thermo-EDTA; P5 Stratec-PAX; S4 Stratec EDTA+PAX; S4 Promega + PAX). In case of S4, only five out of the eight extraction methods yielded sufficient libraries for sequencing. The mean number of sequenced reads (4 libraries per Ion 530 Chip) was 1.75 million reads (+/- 0.87). With only one exception, at least roughly 1 million reads per sample were analyzable. The mean total number of reads per amplicon was 37741.5x.

**Figure 3:**
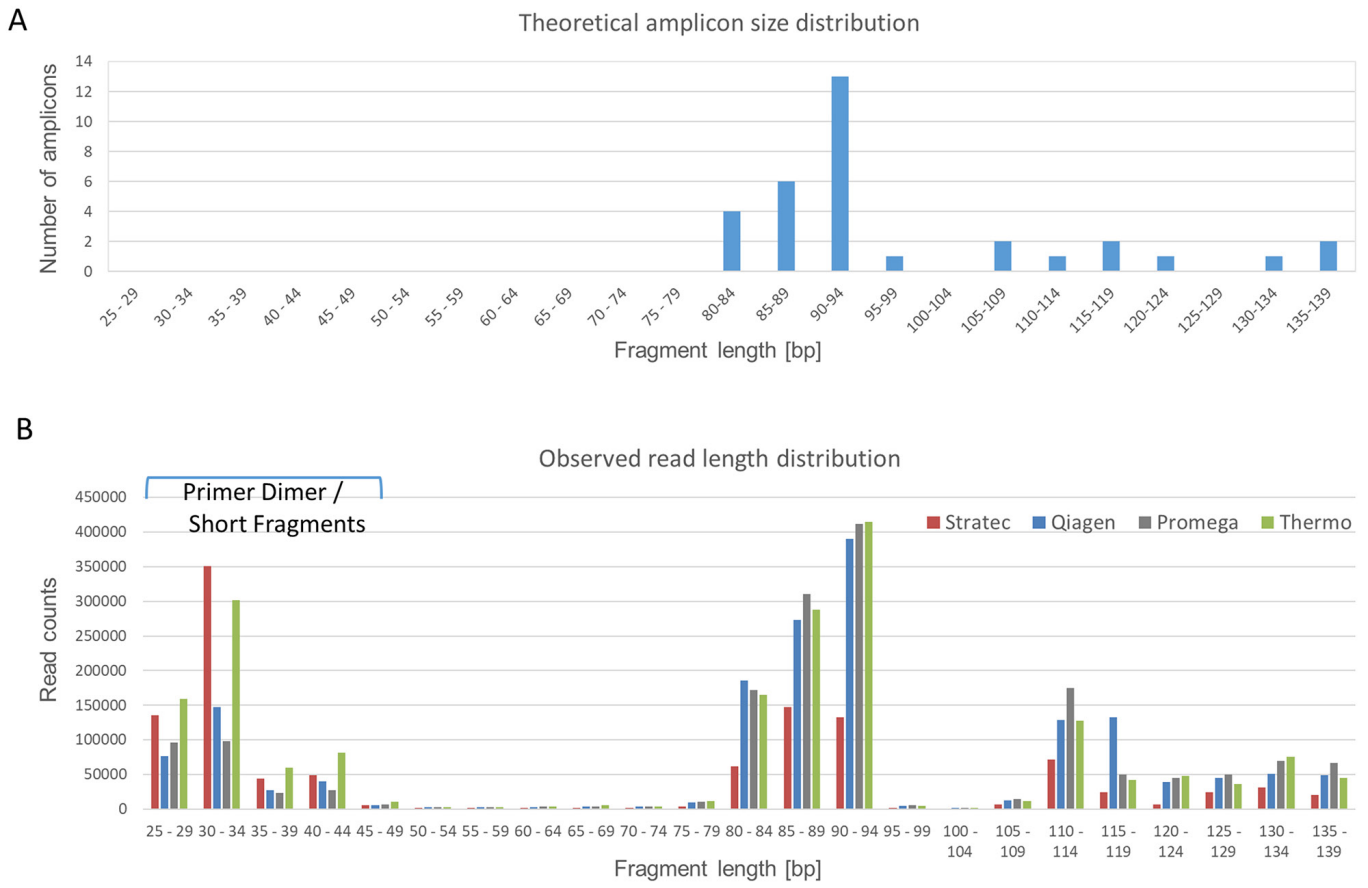
Read Length Distribution. The Oncomine cfDNA Lung panel amplifies 35 different amplicons, ranging in size between 80 and 139 base pairs. **(A)** Theoretical size distribution of the Oncomine cfDNA Lung panel. Amplicons with similar sizes were grouped for better visibility. **(B)** Observed amplicon sizes for sample S2. Colored bars represent the different extraction protocols. Compared to the theoretical size distribution, a group of short fragments representing unincorporated primers is visible. Comparing the different extraction methods, there is a marked difference concerning fragments with short read lengths. The libraries prepared from cfDNA extracted using the protocols from Stratec and Thermo present excess shorter reads (unincorporated primers) with a decrease in target specific reads.

The amplicon lengths of the panel vary between 80 bp and 140 bp, with two clusters of 80-94 bp and 105-139 bp, respectively (Figure 3A). Figure 3B illustrates a typical read length profile obtained from NGS data of the different extraction protocols of case S2. In all cases, there is a party of short reads originating from either primer dimers or short, incomplete sequencing reads. Comparing the distribution patterns, the NGS reads obtained from cfDNA samples derived from Stratec and Thermo extraction protocols show excess of short and incomplete reads. This observation was accompanied by a substantial decline of longer reads when using the Stratec purification method.

### Identification of known mutations

**Figure 4:**
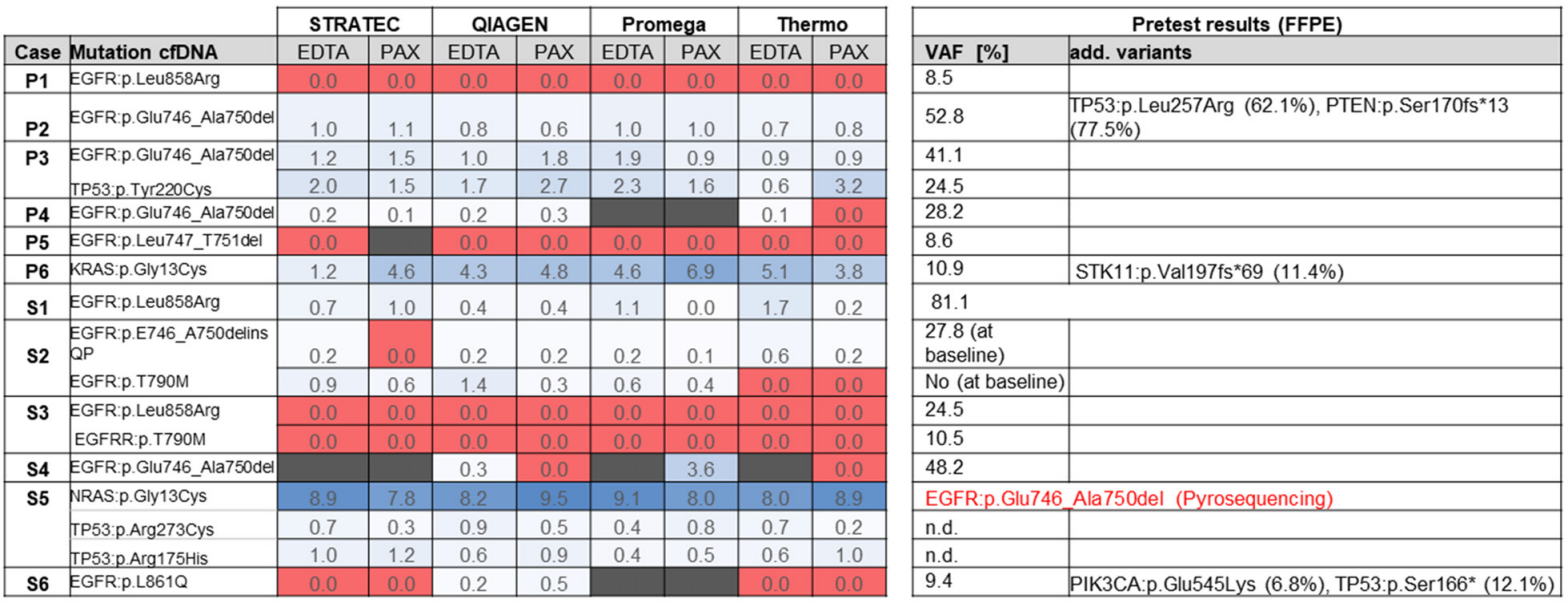
Overview of sequencing results. Numbers represent variant allele frequencies. In sample S5, initial sequencing of the FFPE tumor block prior to TKI therapy identified an EGFR exon 19 deletion, which was not detectable in the cfDNA in progress after TKI.

Figure 4 summarizes the sequencing results of the different cfDNA extractions. In 9 out of 11 samples, the oncogenic driver mutations in *EGFR* or *KRAS *that were identified from FFPE tissue could also be identified in at least one of the cfDNA extractions (sample P1 is not taken into account in this calculation as blood was taken shortly after surgery). In two cases (samples S3 and P5), no mutations were identified using any of the eight extracted cfDNAs. In some cases, the mutations were not called by the variant caller algorithm due to the very low allele frequency, but were identified by manual inspection of the sequencing reads in the IGV browser or a secondary bioinformatics pipeline.

FFPE tissue testing of sample S5 identified an *EGFR* exon 19 deletion at baseline (Table 1). The patient was treated with EGFR kinase inhibitors and radiochemotherapy before onset of disease progression (more detailed description see Table 1). The corresponding blood sample from 2017 now identified an activating *NRAS* mutation along with two additional mutations in *PIK3CA* and *TP53*. These mutations were not examined in the FFPE tissue in 2010. There was unfortunately no original tissue from 2010 left for NGS retesting.

In summary, in 9 out of 11 samples (as mentioned earlier sample P1 was not taken into account), cfDNA was detectable by at least one of the tested extraction methods as judged by detection of oncogenic mutations, correlating to a cfDNA success rate of 82 % (9 out of 11 samples). When considering all samples, tumor-derived ctDNA was detected in 9 out of 12 samples (75%).

For determination of sensitivity of the different extraction protocols, we only considered those nine samples where cfDNA was present and calculated on basis of all mutations present in the collective. The Qiagen and Promega methods correctly identified 25 out of 26 mutations (combined count of all mutations present in the 9 samples multiplied by 2: EDTA and PAXgene) and 21/22 mutations, respectively, thus achieving a sensitivity of 96 %. Using the Thermo Fisher protocol, 19/25 mutations (76 %) were identified, while with the Stratec purification 21/24 mutations (87.5 %) were detected.

### Sequencing artefacts

The tested cfDNA lung panel uses unique molecular identifiers (molecular barcodes) to facilitate differentiation of single molecules present in the plasma from PCR-generated duplicates. The analysis pipeline removes duplicate reads based on the unique molecular barcode and calls variants based on their molecular allelic frequency. In our test cohort, the molecular variant allele frequencies (molVAF) of the primary mutations ranged from 0.1% - 9.45% (Supplementary Table 1). The molecular allele coverage (molCOV) of the primary mutation, e.g. the number of reads showing the mutation after deduplication ranged from two to 277. Thus, the variant calling pipeline reports mutations that can be identified in at least two different reads with unique molecular barcodes.

In our cohort, several variants with low molVAF (0.06 - 3.7%) and molCOV (2-8 molecular reads) were identified by the variant calling pipeline, but were not consistently found throughout the differently extracted cfDNA samples. A closer look for example at an NRAS p.Gly12Asp mutation observed in sample S5 that was found by three different extraction methods (molCOV: 3, 4 and 4 reads) using the IGV browser revealed these calls as false positives based on their presence in short, incomplete reads (compare Supplementary Figure 2 for illustration). Therefore we considered these variants rather as false positive calls instead of true mutations displaying tumor heterogeneity.

Intriguingly, these calls encompassed classical oncogenic driver mutations that can also be found as resistance mutations during EGFR TKI therapy (Supplementary Table 2). For example, in two cases (P3 and S3) low frequency BRAF p.Val600Glu mutations were reported, but only observed in one of the eight analyzed extraction samples. Similarly, a KRAS p.Gly13Ser mutation was called in six different extractions originating from three different individuals.

The difficulty in interpreting these low frequency variant calls can be highlighted by sample S3. The sequencing results of the different cfDNA extractions revealed up to eight mutations with very low molVAFs in the absence of the primary EFGR p.Leu858Arg mutation. While most of these variants were only identified once, two mutations in TP53 (p.Tyr220Cys and p.Arg273His) were found in almost all extractions. Both mutations were not detected by tissue testing. These two TP53 mutations were also observed in samples P3 (p.Tyr220Cys) and S5 (p.Arg273His), but in this case the TP53 p.Tyr220Cys of sample P3 was verified in tissue and the TP53 p.Arg273His was at least found in all sequenced libraries of sample S5.

**Figure 5:**
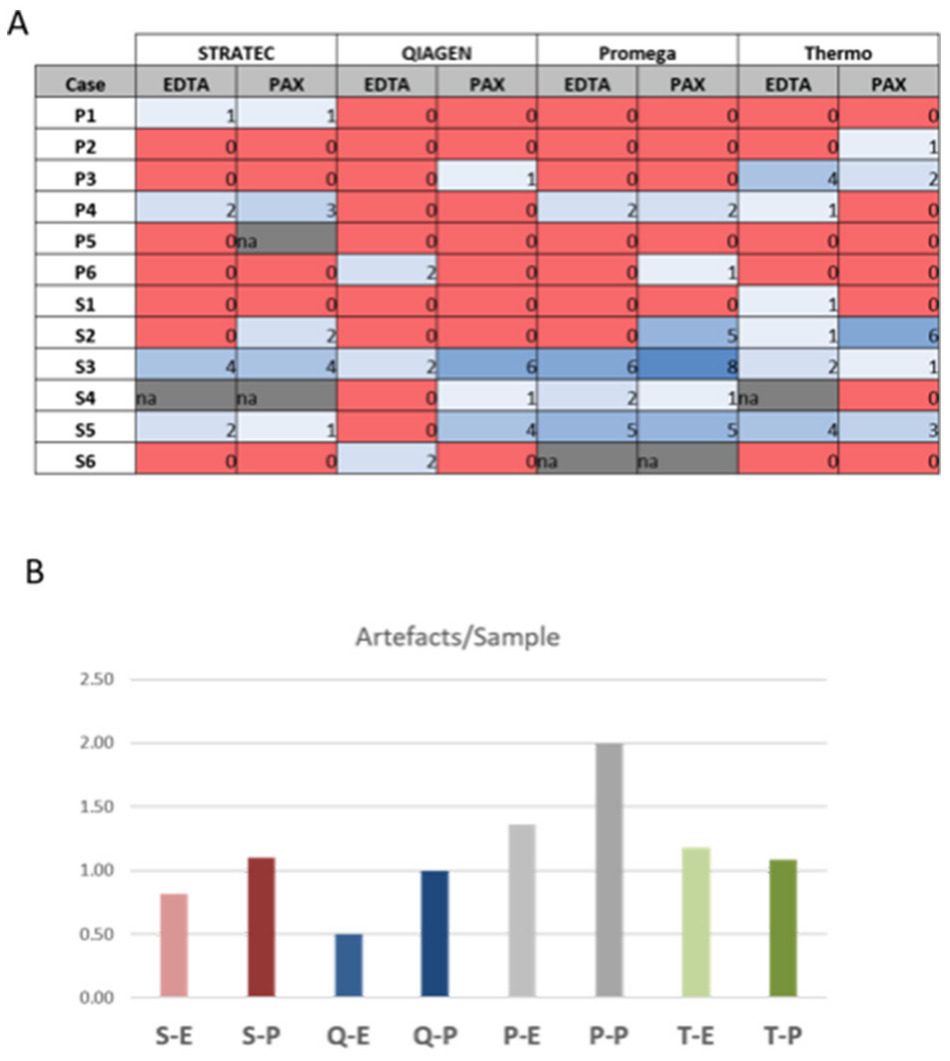
Sequencing artefacts. Numbers represent sum of artefacts identified in each sequencing. Note the slightly higher count of artefacts in PAXgene versus EDTA tubes, but differences are not statistically significant (p=0.266, students t-test). S-E: Stratec-EDTA, S-P: Stratec-PAXgene, Q-E: Qiagen-EDTA, Q-P: Qiagen-PAXgene, P-E: Promega-EDTA, P-P: Promega-PAXgene, T-E: Thermo-EDTA, T-P: Thermo-PAXgene.

Figure 5 and Supplementary Table 2 summarize putative false-positive variants. While in most samples no or up to two false-positive variants were found, in three samples (S2, S3 and S5), eight of these dubious variants were observed. As highlighted in Figure 5B, we found a slightly higher number of false-positive calls in plasma samples that were stored in PAXgene tubes compared to EDTA tubes, but the differences were not statistically significant (p=0.229, paired t-test; p=0.3388; Wilcoxon test). Thus, care should be taken when using plasma-stabilizing tubes.

In order to prove our assumptions on artificially generated mutations we tested some of those samples that provided enough cfDNA after sequencing and showing a *KRAS*, *NRAS* or *BRAF* mutation with a clamped real-time PCR assay. The samples tested were all negative for the suspected mutations in codon 12 and 13 in the *KRAS* gene, for codon 12, 13 in the *NRAS* gene and for the V600E mutation in BRAF. We also tested some eluates of P6 and S5 as they were tested positive for KRAS G13C in tissue and for NRAS G13C in all 8 cfDNA eluates with the UMI-based assay, respectively. All eluates tested were positive for those mutations in the real-time assay as well. A detailed information of tested eluates and results are found in Supplementary Figure 3 and Supplementary Table 3.

## Discussion

Sufficient extraction of cfDNA from plasma of patients suffering from NSCLC is challenging merely because cfDNA concentrations in this context are seriously low anyway [5, 7]. Therefore, no matter how thorough and abundant extraction methods might be the yield of cfDNA is generally borderline. Consequently, we did not expect striking differences on first insight of results but a close careful second look focusing on the quantity but especially on the quality of cfDNA showed differences concerning mutation detection rates. Furthermore as batch-to-batch variability of plasma drawn at different time points during disease progression limits its comparability due to changes in plasma cfDNA levels, we collected up to 60 ml of whole blood from each patients for 8 extractions out of plasma drawn at one time point. We decided to extract plasma at the same time point for EDTA and PAXgene tubes.

The observed overall sensitivity of the liquid biopsy testing in our study was 82 % which is in the typical range reported in the literature, still limiting the capability of liquid biopsy to replace tissue based testing [14, 18]. Comparing the different extraction protocols, the Qiagen minElute assay achieved the highest sensitivity resultingin95.5%, when only considering plasma samples with detectable amounts of ctDNA).

One sample was excluded from this calculation as the patient was operated a week before peripheral blood was taken. Hence the high DNA yield observed for that sample together with the fact that the primary *EGFR* (or any other) mutation could be identified was very likely owed to that circumstance.

Due to the fact that cfDNA concentrations are seriously low and all might contain only a few ctDNA molecules, one may not neglect that any remaining eluted cfDNA that could contain mutated molecules and is excluded from further analysis results into false negative results. One mentionable advantage of the Qiagen minElute extraction method could be that elution volumes can be minimized down to 15 µl meaning that all extracted cfDNA could be used if a sequencing reaction with the Oncomine cfDNA panel only runs once (maximum input of cfDNA: 13 µl). The other extraction methods we evaluated in our study perform a minimum elution volume of 40 µl, 45 µl and 70 µl for the KingFisher instrument, the Maxwell RSC and the Stratec System, respectively. To compare extraction capacities in this study we kept the elution volume of Qiagen to a minimum of 40 µl.

The lower sensitivity of the Stratec and Thermo protocols might be explained by the difference in read length profiles. Our data have shown a decline in target specific reads and a higher amount of short fragments most likely representing primer dimers for both assays.

Of note, in sample S2 both extractions from Thermo detected the primary activating EGFR exon 19 deletion but failed to detect the clinically relevant EGFR p.T790M mutation (read coverage 16806x / 24303x for PAXgene and EDTA, respectively).

### Sequencing artefacts

Due to the low abundance of cfDNA and corresponding low VAF, all methods to detect variants at 0.1% are in risk of identifying false positive variants.

We observed minimal but statistically highly significant differences in cfDNA yields between blood samples stored in EDTA or PAXgene tubes (p=0.000097, paired t-test). The study design concentrated primarily on a possible influence of the stabilizing agents on the sequencing results and not their stabilizing properties, which has been shown in previous studies [19]. While we did not record a decline in amplification efficiencies between EDTA and PAX, we noted a difference in the number of putative sequencing artefacts that was statistically not significant likely due to the small test size. A previous report by Yuhua et al reported a higher number of non-*EGFR* variants observed in samples analyzed using the Oncomine cfDNA Lung assay compared to AmpliSeq generated libraries [20]. The authors also describe oncogenic hotspot variants in *KRAS*, *BRAF* or *NRAS* in samples with primary *EGFR* mutations that might develop during therapy. Several studies have reported on co-occurring *KRAS* and *EGFR* mutations in liquid biopsies that were undetectable in tissue biopsies. Pathway bypass mutations as resistance mechanism have been observed, including for example BRAF p.Val600Glu mutations [21]. However, the biological origin of this mutation is unclear. Deamination processes and BRAF p.Val600Glu mutations in benign naevi are examples that low frequency variants can be present in cfDNA also from healthy individuals [22]. Furthermore, previous studies showed a low concordance level between commercial tissue-based NGS testing and cfDNA analytics [23]. These differences might partially be explained by tumor heterogeneity. This could be the case with sample S5: The originally detected EGFR Exon 19 deletion p.Glu746_Ala750del was not detected in any of the eight extractions but instead we found an NRAS p.Gly13Cys mutation in all extractions. The molecular mutant allelic coverages were all over 100 in all six-sequenced extraction of sample S5. Furthermore this mutation was confirmed by a clamped real-time assay, which was performed on four remaining cfDNA eluates of this patient (Supplementary Figure 3, Supplementary Table 3). Whether the *NRAS* mutation derives from a new lesion or is as a matter of fact a result of possible tumor heterogeneity can be hardly assessed confidently from blood as one cannot determine the location where the mutated fraction in the cfDNA came from. Unfortunately we were not able to gain any more tissue to further explore this issue. On the other hand, the random distribution of discordant variants in our comparative analysis with eight independent sequencings from identical plasma samples, we believe that in the other samples showing these hotspot mutations are rather false positives considering also their low molecular allele coverage and frequency (Supplementary Figure 4).

In summary, the Qiagen minElute shows advantages in quality yield of DNA mainly concerning read length compared to the Stratec and Thermo protocols. We observed a trend that the Qiagen protocol results in a lower number of false-positive artefacts but the sample/mutation number was too small for being statistically relevant. The potential of a minimization of elution volumes down to 15 µl with the Qiagen Assay could be an interesting issue that is worth further investigation. The Oncomine cfDNA Lung panel has a high sensitivity with the advantage of high cfDNA input into one reaction (13 µl) that facilitates detection of oncogenic driver mutations and resistance mechanism from liquid biopsies of lung cancer patients. Pre-analytical steps including the choice of blood tubes and cfDNA extraction method are important to maximize efficiency of liquid biopsy testing. In clinical practice a threshold of 10 allelic reads should be considered to maximize specificity especially when single nucleotide variants (SNV), need to be identified undoubtedly.

A.S. and V.E. conducted the experiments, analyzed results and wrote the manuscript. T.M. and Al.S. supervised the manuscript. A.S. collected samples and blood and extracted cfDNA from all samples with four different extraction methods. J.K; D.M. and T.G.B. provided clinical data, S.S.-F. provided histopathological data, A.E. and O.L. run real-time PCR tests

## Materials and Methods

### Patient cohort

During April 2017 and August 2017, peripheral whole blood was collected from 12 non-small cell lung cancer patients during ongoing treatment, five before initiation of first line systemic therapy (Samples P2-P5), one after post-surgery (P1) and six patients after first line systemic therapy (S1-S6). All patients harboured an activating mutation either in the *EGFR* or *KRAS* gene (Table 1). The activating mutation had already been analysed from FFPE tissue during routine diagnostics prior to blood sampling using targeted sequencing with the AmpliSeq Colon Lung Panel v2 on the Ion Torrent S5XL instrument (Thermo Scientific, Waltham, USA). Only Sample S5 was analyzed by pyrosequencing as NGS was not practiced in our institute at the time point of first diagnosis in 2010 and no more tissue or DNA was left to retest the latter with NGS methods.

Informed consent was obtained from each patient with protocols approved by an ethical committee.

### Plasma samples

As batch-to-batch variability of plasma drawn at different time points during disease progression can occur and may limit its comparability due to changes in plasma cfDNA levels, we collected 60 ml blood at one time point from each patient. Two 30 ml batches of whole blood were taken and collected in EDTA or PAXgene tubes (Qiagen, Hilden, Germany), respectively. Blood samples were immediately centrifuged at 1300x g for 13 min [16]. Plasma was transferred to new tubes and centrifuged for 12 min at 16.000x g, plasma drawn from PAXgene tubes were centrifuged at room temperature whereas EDTA plasma was centrifuged at 4°C [16]. Clear supernatants were stored short term at -20°C or extracted immediately.

### cfDNA extraction and quantification

Frozen plasma samples were thawed and purified by filtration (Minisart Syringe filter, Sartorius, Göttingen, Germany). cfDNA was extracted from 4 ml plasma by using each of the following methods: InviMag® Free Circulating DNA Kit, (Stratec, Berlin, Germany), minElute ccfDNA extraction kit (Qiagen, Hilden, Germany), Maxwell RSC ccfDNA extraction kit (Promega, Madison, USA) and MagMax cfDNA extraction (Thermo Scientific, Waltham, USA) on a KingFisher instrument according to the instruction of the manufacturer. Elution volumes ranged between 36 and 70 µl (for detailed description see Table 2). cfDNA quantification was performed by a fluorescent assay using the Qubit 3.0 Fluorometer (Thermo Scientific, Waltham, USA).

Qualitative assessment of cfDNA was performed on a TapeStation 2200 (Agilent Technologies, Santa Clara, USA) using up to 2 µl cfDNA on a high-sensitivity D1000 tape according to manufacturer instructions.

### NGS library preparation

For this study, we used the Oncomine Lung cfDNA panel (Thermo Scientific, Waltham, USA) which has a theoretical limit of detection of 0.1% VAF if using at least 20 ng cfDNA input.

The panel comprises 35 amplicons covering clinically actionable hotspot mutations from 11 genes using the proprietary TagSequencing technology. From each extraction, NGS sequencing libraries using the Oncomine cfDNA Lung panel were prepared, resulting in 96 libraries overall. We used the maximum possible input volume of 13 µl for NGS library generation using the TagSequencing protocol.

This library preparation method incorporates unique identifiers (molecular barcodes) in a two-cycle PCR reaction allowing deduplication of sequenced reads for increased sensitivity. Following purification with AmPureXP Beads (Agilent, Santa Clara, USA), a second PCR (18 cycles) is used for library preparation. After two further rounds of purification using AmpureXP beads, the final library is quantified using the Ion Library quantification kit (Thermo Scientific, Waltham, USA). In total, 6 out of 96 libraries did not match quality criteria and were excluded from further analysis. On average, 4-5 libraries were pooled on an Ion 530 chip. Following template preparation on an Ion Chef, the chip was sequenced on an Ion S5XL sequencer using 200 bp chemistry and 500 flows.

### Mutation analysis

Sequence alignment and variant calling was performed using plugins in Torrent Suite version 5.6 and IonReporter 5.2 / 5.6. Data was analyzed with the IGV browser.

### Real-time PCR testing

DNA extracts (2 µl) were tested with the beta version of LightMix kit 40-0654-64 NRAS-KRAS, comprising of a multiplex pre-amplification of the gene regions followed by clamped-probe wild type sequence suppressing amplification of the four codon regions 12/13, 59/61, 117 and 146 in NRAS and KRAS, and identification of mutations by running a melting curve, using a Roche (Mannheim, Germany) cobas z 480 analyzer. KRAS mutation results were confirmed using LightMix kit 40-0416-09 KRAS 12/13, BRAF mutations were identified with the LightMix kit 40-0406-96 BRAF, following the kit instructions.

## SUPPLEMENTARY MATERIALS FIGURES AND TABLES







## References

[1] Santarpia M , Altavilla G , Salazar MF , Magri I , Pettineo G , Benecchi S , Rosell R . Tyrosine kinase inhibitors for non-small-cell lung cancer: finding patients who will be responsive. Expert Rev Respir Med. 2011; 5:413–24. 10.1586/ers.11.27. 21702662

[2] Thomas A , Liu SV , Subramaniam DS , Giaccone G . Refining the treatment of NSCLC according to histological and molecular subtypes. Nat Rev Clin Oncol. 2015; 12:511–26. 10.1038/nrclinonc.2015.90. 25963091

[3] Diaz LA Jr , Bardelli A . Liquid biopsies: genotyping circulating tumor DNA. J Clin Oncol. 2014; 32:579–86. 10.1200/JCO.2012.45.2011. 24449238 PMC4820760

[4] Schwarzenbach H , Hoon DS , Pantel K . Cell-free nucleic acids as biomarkers in cancer patients. Nat Rev Cancer. 2011; 11:426–37. 10.1038/nrc3066. 21562580

[5] Bettegowda C , Sausen M , Leary RJ , et al. Detection of circulating tumor DNA in early- and late-stage human malignancies. Sci Transl Med. 2014; 6:224ra224–224ra224. 10.1126/scitranslmed.3007094. 24553385 PMC4017867

[6] van Dessel LF , Vitale SR , Helmijr JC , Wilting SM , van der Vlugt-Daane M , Oomen-de Hoop E , Sleijfer S , Martens JW , Jansen MP , Lolkema MP . High-throughput isolation of circulating tumor DNA: a comparison of automated platforms. Mol Oncol. 2019; 13:392–402. 10.1002/1878-0261.12415. 30516338 PMC6360376

[7] van Ginkel JH , van den Broek DA , van Kuik J , Linders D , de Weger R , Willems SM , Huibers MM . Preanalytical blood sample workup for cell-free DNA analysis using Droplet Digital PCR for future molecular cancer diagnostics. Cancer Med. 2017; 6:2297–307. 10.1002/cam4.1184. 28940814 PMC5633557

[8] Cook L , Starr K , Boonyaratanakornkit J , Hayden R , Sam SS , Caliendo AM . Does Size Matter? Comparison of Extraction Yields for Different-Sized DNA Fragments by Seven Different Routine and Four New Circulating Cell-Free Extraction Methods. J Clin Microbiol. 2018; 56:e01061–18. 10.1128/JCM.01061-18. 30282788 PMC6258844

[9] Volckmar AL , Sültmann H , Riediger A , Fioretos T , Schirmacher P , Endris V , Stenzinger A , Dietz S . A field guide for cancer diagnostics using cell-free DNA: from principles to practice and clinical applications. Genes Chromosomes Cancer. 2018; 57:123–39. 10.1002/gcc.22517. 29205637

[10] Newman AM , Lovejoy AF , Klass DM , Kurtz DM , Chabon JJ , Scherer F , Stehr H , Liu CL , Bratman SV , Say C , Zhou L , Carter JN , West RB , et al. Integrated digital error suppression for improved detection of circulating tumor DNA. Nat Biotechnol. 2016; 34:547–55. 10.1038/nbt.3520. 27018799 PMC4907374

[11] Murtaza M , Dawson SJ , Tsui DW , Gale D , Forshew T , Piskorz AM , Parkinson C , Chin SF , Kingsbury Z , Wong AS , Marass F , Humphray S , Hadfield J , et al. Non-invasive analysis of acquired resistance to cancer therapy by sequencing of plasma DNA. Nature. 2013; 497:108–12. 10.1038/nature12065. 23563269

[12] Thierry AR , Mouliere F , El Messaoudi S , Mollevi C , Lopez-Crapez E , Rolet F , Gillet B , Gongora C , Dechelotte P , Robert B , Del Rio M , Lamy PJ , Bibeau F , et al. Clinical validation of the detection of KRAS and BRAF mutations from circulating tumor DNA. Nat Med. 2014; 20:430–35. 10.1038/nm.3511. 24658074

[13] Zonta E , Garlan F , Pécuchet N , Perez-Toralla K , Caen O , Milbury C , Didelot A , Fabre E , Blons H , Laurent-Puig P , Taly V . Multiplex Detection of Rare Mutations by Picoliter Droplet Based Digital PCR: Sensitivity and Specificity Considerations. PLoS One. 2016; 11:e0159094. 10.1371/journal.pone.0159094. 27416070 PMC4945036

[14] Vollbrecht C , Lehmann A , Lenze D , Hummel M . Validation and comparison of two NGS assays for the detection of EGFR T790M resistance mutation in liquid biopsies of NSCLC patients. Oncotarget. 2018; 9:18529–39. 10.18632/oncotarget.24908. 29719623 PMC5915090

[15] Li BT , Janku F , Jung B , Hou C , Madwani K , Alden R , Razavi P , Reis-Filho JS , Shen R , Isbell JM , Blocker AW , Eattock N , Gnerre S , et al. Ultra-deep next-generation sequencing of plasma cell-free DNA in patients with advanced lung cancers: results from the Actionable Genome Consortium. Ann Oncol. 2019; 30:597–603. 10.1093/annonc/mdz046. 30891595 PMC6503621

[16] Sorber L , Zwaenepoel K , Deschoolmeester V , Roeyen G , Lardon F , Rolfo C , Pauwels P . A Comparison of Cell-Free DNA Isolation Kits: Isolation and Quantification of Cell-Free DNA in Plasma. J Mol Diagn. 2017; 19:162–68. 10.1016/j.jmoldx.2016.09.009. 27865784

[17] Risberg B , Tsui DW , Biggs H , Ruiz-Valdepenas Martin de Almagro A , Dawson SJ , Hodgkin C , Jones L , Parkinson C , Piskorz A , Marass F , Chandrananda D , Moore E , Morris J , et al. Effects of Collection and Processing Procedures on Plasma Circulating Cell-Free DNA from Cancer Patients. J Mol Diagn. 2018; 20:883–92. 10.1016/j.jmoldx.2018.07.005. 30165204 PMC6197164

[18] Oxnard GR , Thress KS , Alden RS , Lawrance R , Paweletz CP , Cantarini M , Yang JC , Barrett JC , Jänne PA . Association Between Plasma Genotyping and Outcomes of Treatment With Osimertinib (AZD9291) in Advanced Non-Small-Cell Lung Cancer. J Clin Oncol. 2016; 34:3375–82. 10.1200/JCO.2016.66.7162. 27354477 PMC5035123

[19] Alidousty C , Brandes D , Heydt C , Wagener S , Wittersheim M , Schäfer SC , Holz B , Merkelbach-Bruse S , Büttner R , Fassunke J , Schultheis AM . Comparison of Blood Collection Tubes from Three Different Manufacturers for the Collection of Cell-Free DNA for Liquid Biopsy Mutation Testing. J Mol Diagn. 2017; 19:801–04. 10.1016/j.jmoldx.2017.06.004. 28732213

[20] Yuhua L , Yee JM , Min T . Targeted Next-generation Sequencing of Circulating Biomarkers in Non-Small Cell Lung Cancer (NSCLC). IRCSet Conference. http://ircset.org/anand/2017papers/IRC-SET_2017_paper_S4-3.pdf.

[21] Huang L , Fu L . Mechanisms of resistance to EGFR tyrosine kinase inhibitors. Acta Pharm Sin B. 2015; 5:390–401. 10.1016/j.apsb.2015.07.001. 26579470 PMC4629442

[22] Pollock PM , Harper UL , Hansen KS , Yudt LM , Stark M , Robbins CM , Moses TY , Hostetter G , Wagner U , Kakareka J , Salem G , Pohida T , Heenan P , et al. High frequency of BRAF mutations in nevi. Nat Genet. 2003; 33:19–20. 10.1038/ng1054. 12447372

[23] Chae YK , Davis AA , Carneiro BA , Chandra S , Mohindra N , Kalyan A , Kaplan J , Matsangou M , Pai S , Costa R , Jovanovic B , Cristofanilli M , Platanias LC , Giles FJ . Concordance between genomic alterations assessed by next-generation sequencing in tumor tissue or circulating cell-free DNA. Oncotarget. 2016; 7:65364–73. 10.18632/oncotarget.11692. 27588476 PMC5323161

