# Supplementary material for: Comparison of different semi-automated cfDNA extraction methods in combination with UMI-based targeted sequencing
Source: Oncotarget. 2019 Oct 1;10(55):5690–702. doi: 10.18632/oncotarget.27183 (PMC6779285; doi:10.18632/oncotarget.27183)
Supplement: Supplementary file 1 [file oncotarget-10-5690-s001.pdf]

Comparison of different semi-automated cfDNA extraction methods in combination with UMI-based targeted sequencing

SUPPMENTARY MATERIALS

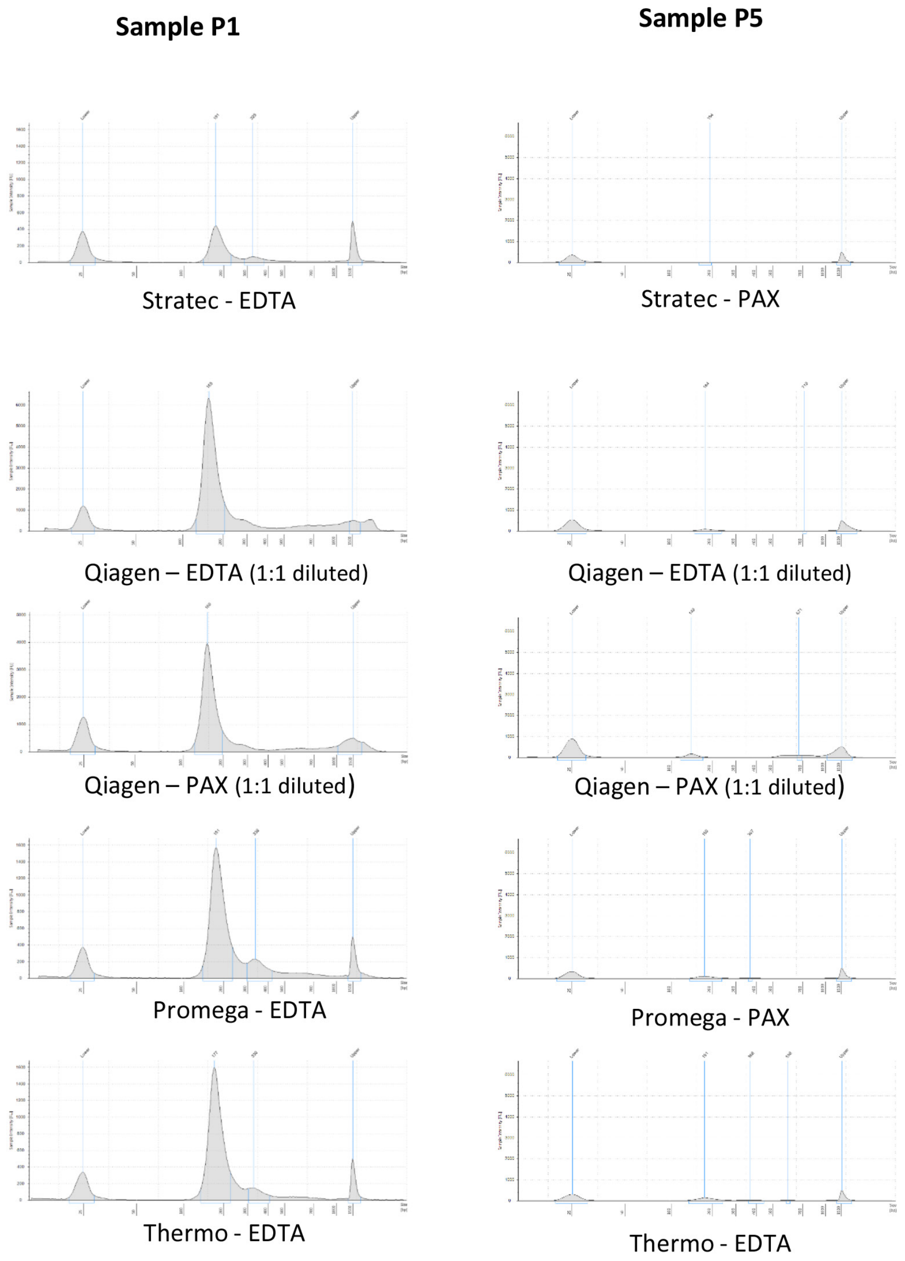

Supplementary Figure 1: Assessment of cfDNA quality by fragment length analysis. *Left panel***:** cfDNA profile comparision between the different extraction methods for sample P1. The profiles (concentration from 0.8-7ng/µl) show the typical patterns for cfDNA with peaks at 175bp and 353bp. The calculated concentrations from the TapeStation correspond approximately to the measured values by QuBit. *Right panel*: cfDNA profiles for sample P5 (concentration range 0.057-0.5ng/µl). Peak intensities for most other samples not illustrated here show similar patterns.

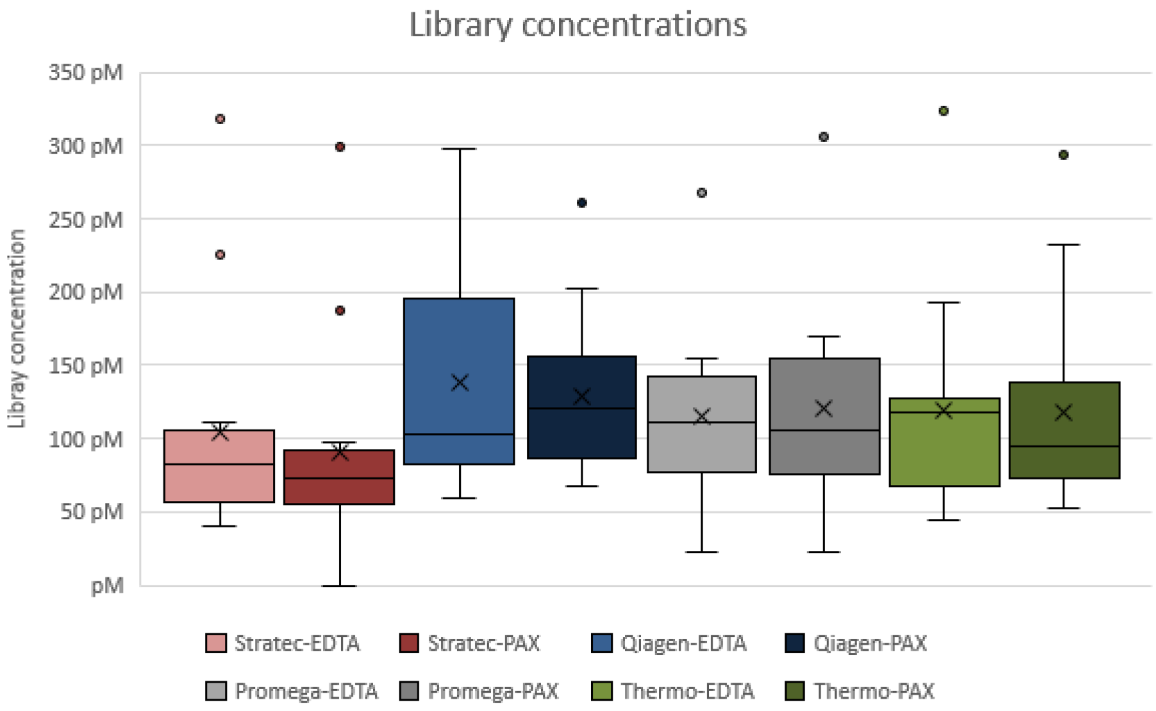

Supplementary Figure 2: Box and Whisker plot comparing the library concentrations measured by qPCR for all samples. The library concentrations from Stratec are significantly lower compared to Qiagen or Thermo (paired t-test: $p=0.0024$ and $p=0.026$), but not to Promega (paired t-test: $p=0.118$).

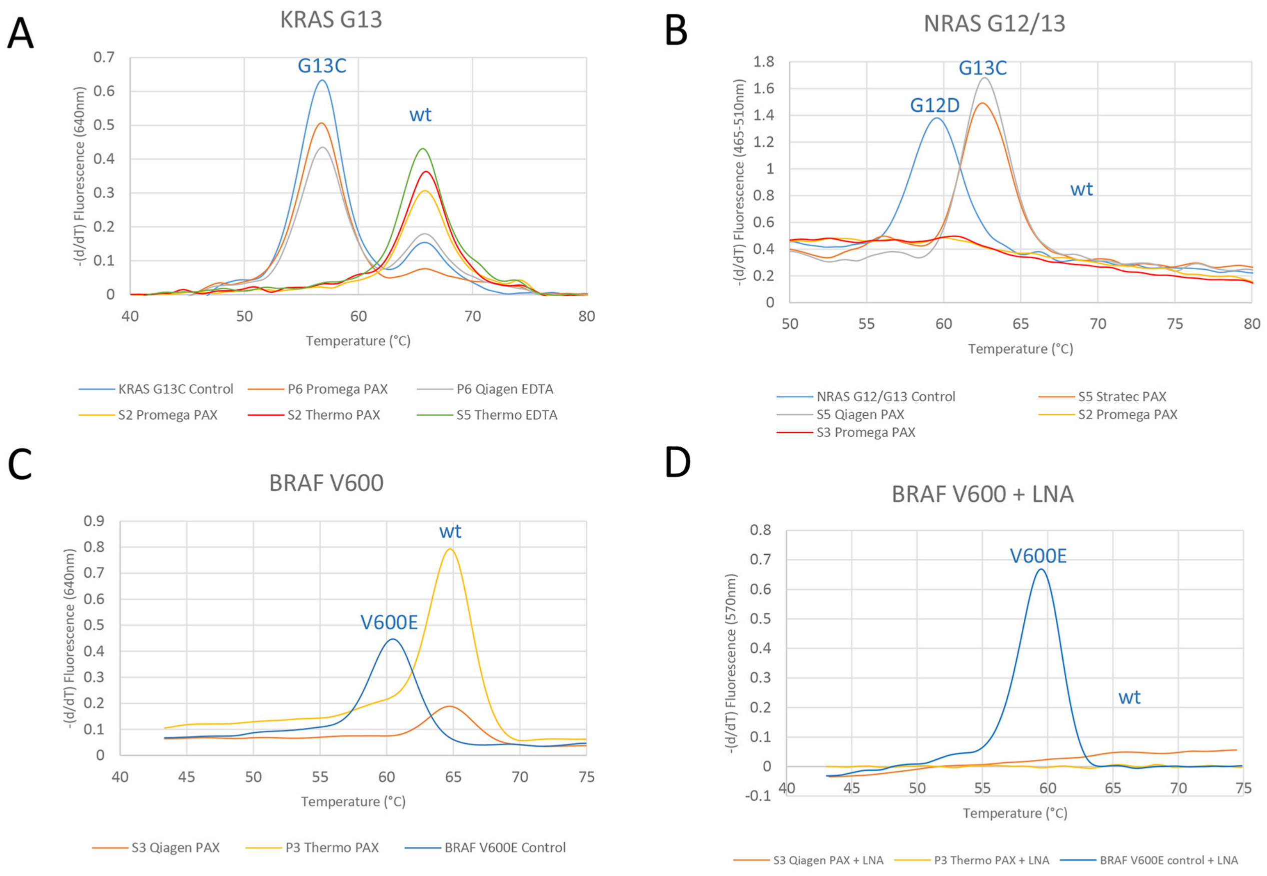

Supplementary Figure 3: Validation of NGS results by high-sensitive clamped real-time assays. (A) KRAS G12/G13 assay. The peak at 56.2°C corresponds to the KRAS p.G13C mutation, while the peak at 65°C represents the wildtype situation. Sample P6 with a KRAS p.G13C mutation detected by NGS in all analyzed extractions shows a clear peak at 56.2°C, confirming NGS results. In sample S2 (Thermo/PAXgene) and S5 (Thermo/EDTA), a KRAS p.G13S was detected by NGS with low molecular allele coverage. In the clamped assay, only the peak at 65°C – representing the wildtype- is visible, unmasking these mutations as artefacts. **(B)** Clamped real-time assay for NRAS G12/G13 mutations. The peak at ~59°C depicts the NRAS p.G12D mutation, while the peak at ~62°C marks a p.G13C mutation that was identified in sample S5. In sample S2 (Promega/EDTA) and S3 (Promega/PAXgene), no peaks are visible indicating wildtype (wildtype peak completely suppressed by LNA). **(C and D)** BRAF V600 assay without (C) and with LNA (D). In the presence of LNA, the wildtype peak is suppressed. Both tested samples that showed low-frequency BRAF V600E mutations by NGS are negative by the real-time assay.

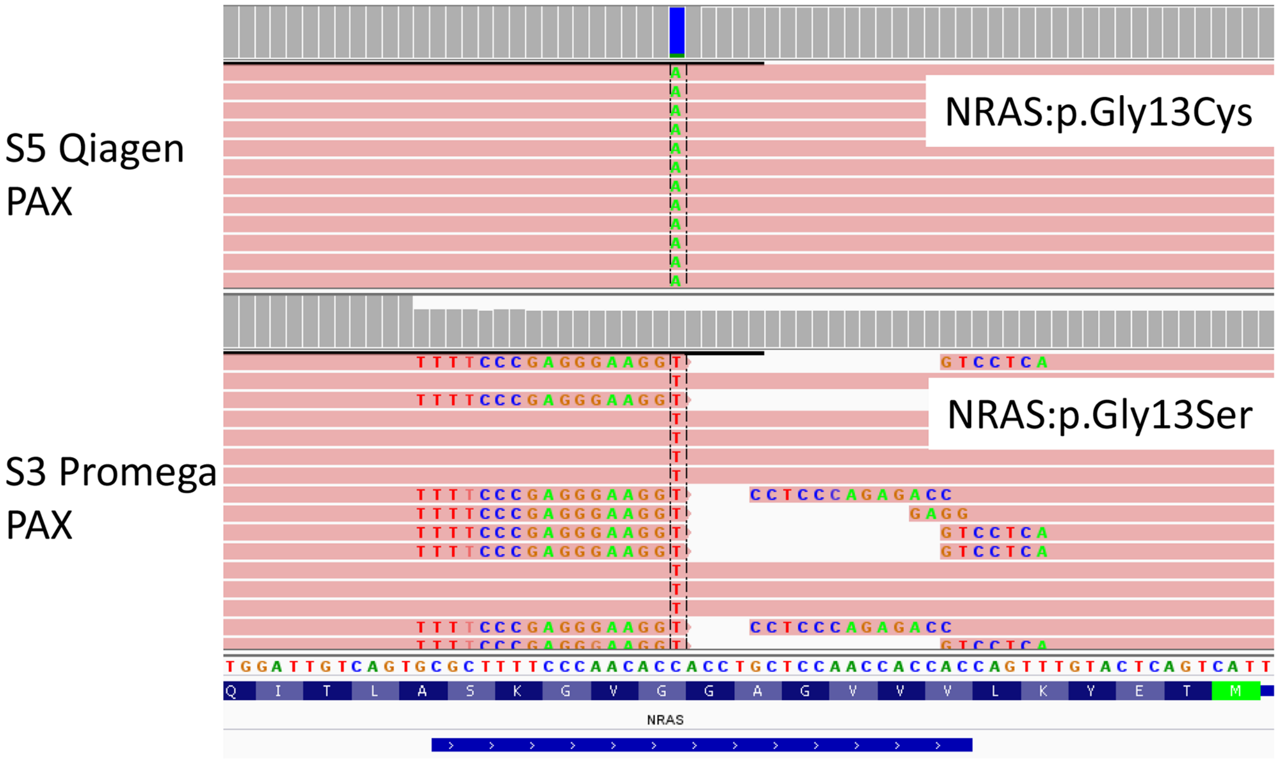

Supplementary Figure 4: Visualization of artefectual variants by IGV browser. Note the presence of the false-positive base exchange at softclipped positions in incomplete reads.

Supplementary Table 1: Mutations identified in the different cfDNA extractions.

See Supplementary File 1

Supplementary Table 2: Overview of sequencing artefacts found in cfDNA.

See Supplementary File 2

Supplementary Table 3: Validation of NGS results

Sample ID	plasma	cfKit	mutation real	expected artefact	Real Time PCR
P1	edta	stratec		KRAS G13S	neg.
P1	pax	stratec		KRAS G13S	neg.
P1	pax	thermo		wt	neg.
P6	edta	stratec	KRAS G13C		neg.
P6	edta	qiagen	KRAS G13C		pos.
P6	pax	promega	KRAS G13C	KRAS G13S	pos. for G13C
P3	pax	qiagen		NRAS G12fs*	neg.
S2	pax	promega		KRAS G13S	neg.
S2	pax	thermo		KRAS G13C	neg.
S3	pax	promega		NRAS G13S	neg.
S5	edta	stratec	NRAS G13C		pos.
S5	pax	stratec	NRAS G13C		pos.
S5	edta	qiagen	NRAS G13C		not tested
S5	pax	qiagen	NRAS G13C		pos.
S5	edta	thermo	NRAS G13C		pos.
P3	pax	thermo		BRAF V600E	neg.
S3	pax	qiagen		BRAF V600E	neg.
